# Reconstruction of Chest Wall by Cryopreserved Sternal Allograft after Resection of Aneurysmal Bone Cyst of Sternum

**DOI:** 10.1155/2017/9135657

**Published:** 2017-02-19

**Authors:** Kambiz Sheikhy, Azizollah Abbasi Dezfouli, Farahnaz Sadegh Beigee

**Affiliations:** Department of Thoracic Surgery, Lung Transplant Research Center, National Research Institute of Tuberculosis and Lung Diseases (NRITLD), Masih Daneshvari Hospital, Shahid Beheshti University of Medical Sciences, Tehran, Iran

## Abstract

A 20-year-old female was referred to our hospital due to deformity and bulging in anterior aspect of chest wall in sternal area. Chest X-ray and CT scan confirmed a large mass with destruction of sternum. Pathologic diagnosis after incisional biopsy was compatible with aneurysmal bone cyst. We resected sternum completely and reconstructed large anterior defect by a cryopreserved sternal allograft. In follow-up of patient there was no unstability of chest wall with good cosmetic result.

## 1. Introduction

Radical resection of sternum is indicated in different situations such as primary or secondary malignant tumors, some rare cases of benign tumors, infection, radio necrosis, and trauma [1]. Aneurysmal bone cyst (ABC) is a rare benign bone tumor that comprises about 2.5% of all bone tumors and is extremely rare in sternum. Complete surgical resection is considered optimal therapy for these patients if feasible [2]. In most cases surgical resection of sternal lesion is not problematic but reconstruction of this part of the chest wall is challenging for most thoracic and reconstructive surgeons. In this group of patients reconstruction of chest wall is very important for prevention of flail chest and ventilation impairment. Cosmetic issues must also be considered [3].

Various techniques have been described but all of them have advantages and disadvantages and at the present time there is no gold standard for managing these defects [4]. There is limited experience with a new method for reconstruction of sternum using cryopreserved sternal allograft.

In this case report we have described a patient with sternal aneurysmal bone cyst that after complete surgical resection of tumor (sternectomy) reconstruction of chest wall was carried with a cryopreserved sternal allograft.

## 2. Case Report

**Figure 1:**
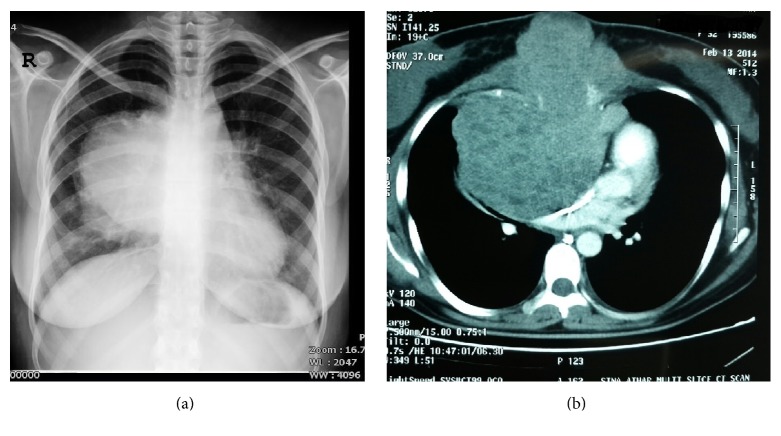
Preoperative chest X-ray (a) and CT scan (b) showing a large mass with multiple areas of low attenuation that has compressed the heart but without obvious invasion to vital organs.

A 20-year-old female patient was referred to our thoracic surgery unit due to deformity and bulging in the anterior part of chest wall in sternal area with mild pain. A tumor was suspected based on the chest X-ray and therefore a chest CT scan was done that revealed a large mass with differential diagnosis of primary sternal tumor in origin or anterior mediastinal tumor with invasion to sternum (Figures 1(a) and 1(b)).

A core needle biopsy was performed with pathologic diagnosis compatible with giant cell tumor. An incisional biopsy was done based on pathologist's recommendation which revealed a highly vascular mass with final pathologic diagnosis of aneurysmal bone cyst.

The best treatment option was deemed to be resection of lesion and due to the size of tumor, total sternectomy was planned.

**Figure 2:**
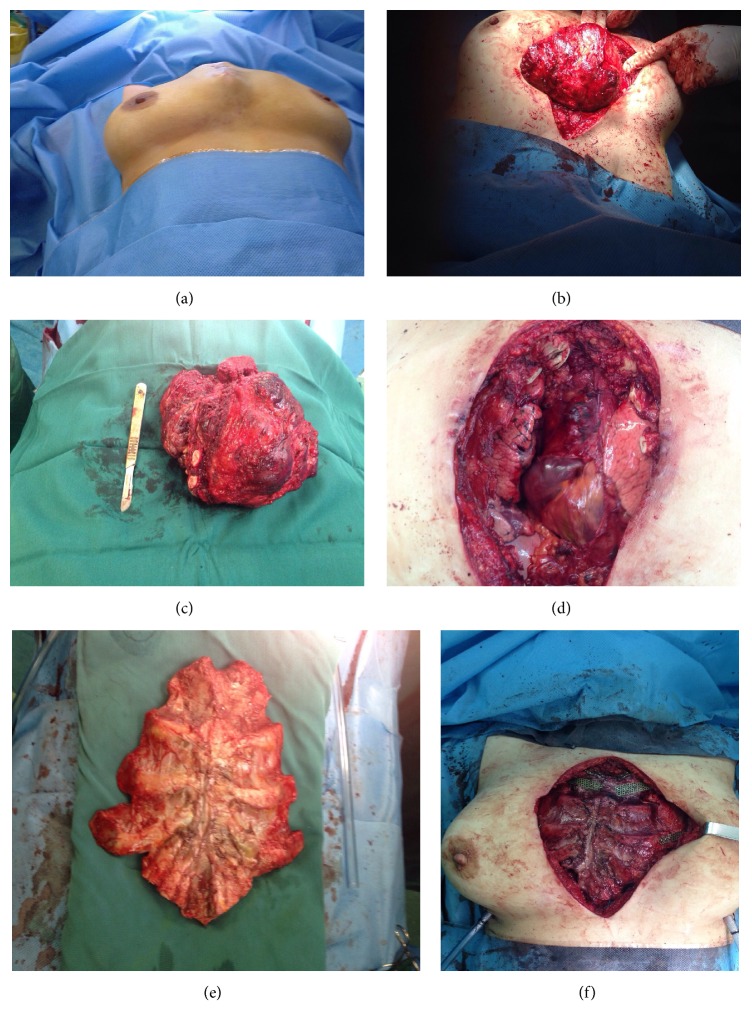
Preoperative picture of patient with a large bulging in anterior mediastinal area (a). At the time of operation a large hemorrhagic mass without obvious invasion to surrounding tissues was discovered (b) that was completely resected along with sternal manubrium (c). Large anterior defect of chest wall (d) was reconstructed by a cryopreserved sternal allograft (e) that was fixed by titanium plates and screw (f).

At the time of surgery a longitudinally elliptical midline incision was performed which included previous biopsy site. There was no infiltration or adhesion of mass to skin and subcutaneous tissue and a well-defined, capsulated but highly vascular mass was dissected from surrounding tissues. Due to large dimension of tumor, pectoralis major muscles were pushed laterally to nearly mid clavicular lines (Figures 2(a) and 2(b)). Thoracic cavity was entered with some difficulty in hemostasis on the left side and all attachments of sternum and ribs were cut 2 centimeters away from tumor. The same steps were repeated on the right side. There was minimal adhesion of tumor to right upper lobe that was lysed easily but dense adhesion to anterior part of pericardium was managed by partial resection of pericardium and repairing the pericardium with PTFE mesh. Due to involvement of manubrium, total sternectomy was achieved by disarticulation of bilateral sternoclavicular joints. Large 20 × 20 cm mass was completely resected and an extensive defect remained in the chest wall (Figures 2(c) and 2(d)). According to previous planning a cryopreserved sternal allograft was considered to repair the defect. Sternal graft was harvested from suitable cadaver in our center and cryopreserved in another tissue bank about one month prior to operation and was finally transferred for transplantation. Graft was tailored gradually and fixed to patient's ribs by titanium mesh plates and screws and exactly fixed to bilateral clavicles (Figures 2(e) and 2(f)). Subsequently covering of the graft by pectoralis muscles was not possible and therefore breast tissue was approximated in midline for covering the graft and skin repairing was performed without any breast disfiguration. Bilateral chest tubes were inserted and wide spectrum antibiotics were administered. After surgery, chest wall was completely stable and there was no need for ventilator support. Chest tubes were removed 3 days after operation and there was no sign and symptom of wound infection. Antibiotics were continued till fifth postoperative day and patient was discharged one week after surgery.

**Figure 3:**
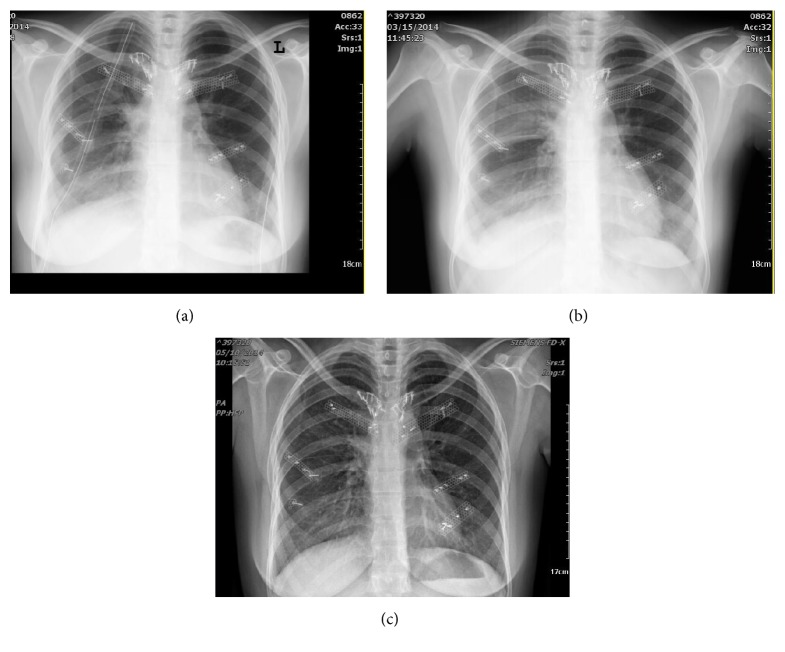
Serial postoperative chest X rays. Early postoperative (a) about two weeks (b) and nearly three months after operation (c).

**Figure 4:**
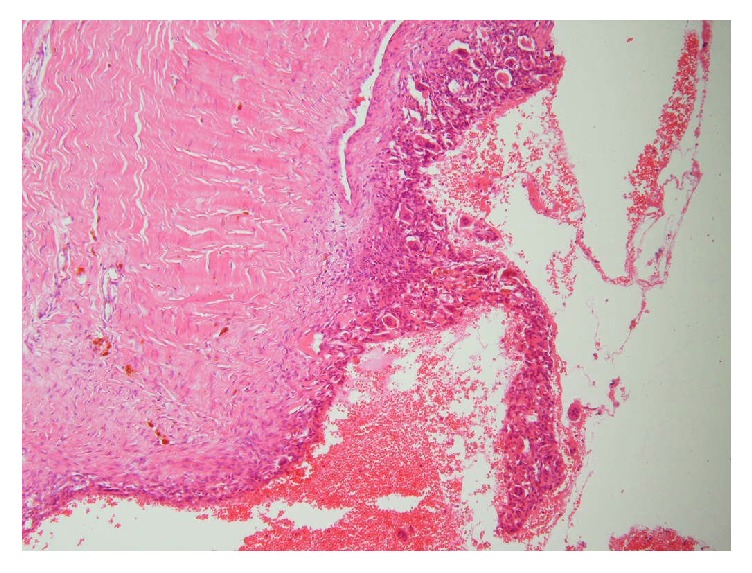
Histopathology (H&A, 200x) showed that the tumor cells exhibited multiple cystic lesion that was filled with red blood cells without endothelial lining surrounded by osteoclast like giant cells.

**Figure 5:**
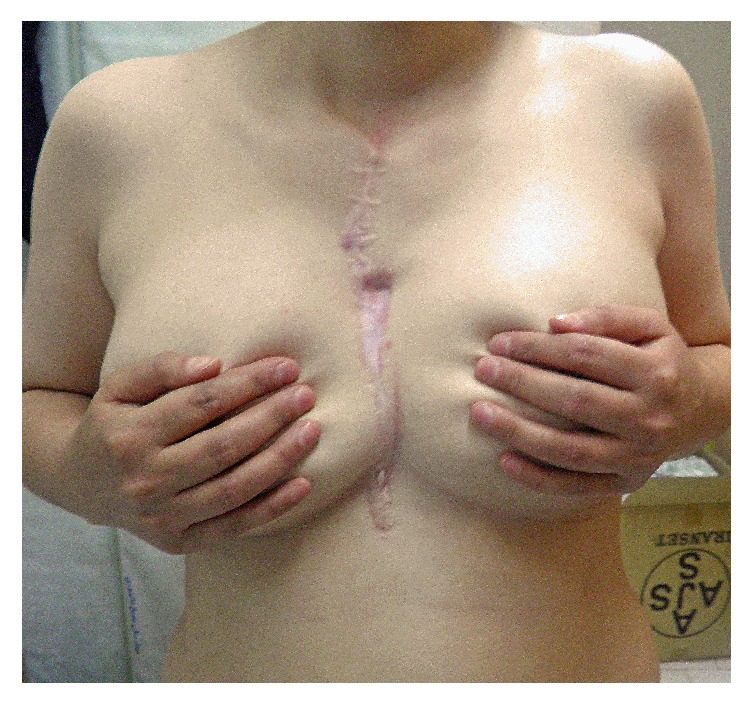
About 18 months after sternal resection there is no evidence of tumoral lesion with small keloid tissue in incisional line.

In serial visits 1, 2, 3, and 12 months after surgery the defect was completely stable with no complicating events. Final pathologic report was compatible with aneurysmal bone cyst (Figure 4). Serial chest X rays of patient have been shown in Figure 3. More than eighteen months after surgery, patient had some keloid tissue in primary incision with no evidence of tumor regrowth (Figure 5).

## 3. Discussion

Primary tumors of sternum are rare and include about 5% of all bony tumors. Benign sternal tumors are extremely rare. Aneurysmal bone cyst with an incidence of 2.5–5% of bone tumors is an extremely rare pathology in sternum and limited number of cases have been reported [2]. Although experience is limited most authors agree with excisional biopsy or even more radical surgery whenever possible, as was in this case. After resection of sternum based on different pathologic processes each surgeon has two key points in his/her mind: reconstruction of bony part of thoracic cage and then soft tissue coverage of the reconstructed site. Most challenging part of operation is selection of the best reconstructive strategy for reconstruction of bony part of anterior chest wall. Reconstruction of soft tissue defect with various types of flaps is less challenging.

Methyl methacrylate mesh or sandwiches, polytetrafluroethylene (PTFE), or polypropylene patches belong to routine thoracic surgical armamentarium and considering advantages and disadvantages of their use, extensive usage has been described. Recently metallic material such as titanium clips or plates and even moldable multiholed plates have been used for stabilization of chest wall [5]. Turna et al. [6] recently described reconstruction of anterior chest wall defect after wide resection of this part with a well-designed patient specific titanium implant. A hydroxyapatitetricalcium-phosphate compound (ceramic) that can be shaped has been introduced for reconstruction [5].

Due to complications related to using prosthetic material such as infection and rigidity, bone grafts have gained considerable attention in reconstruction of chest wall [7]. Ribs can be harvested as autograft from opposite surgical site or can be used as allograft from tissue banks. When chest wall defect is large, as in cases with resected sternal tumors, harvesting bone autograft can result in significant morbidity for patient [8]. A potentially inexhaustible source of bone for reconstruction of chest wall defects is human cadaver. Following the large experiences of orthopedic surgeons for filling bony defects and restoring skeletal continuity, use of cryopreserved allograft bones has recently been successfully introduced in thoracic surgery [5]. The main advantage of bone grafts is their capability of integration with host tissue and good chest wall stability without rejection, infection, and migration. Bone graft also acts as a scaffold for osteoprogenitor cells that will migrate in it and new bone will form [8]. Recently some authors have used cryopreserved sternal bone allograft for a limited number of patients after sternal resection [8–10]. In 2012 Stella et al. reported a single case of allogenic sternal transplant after sternectomy for metastatic ovarian cancer with acceptable results [9]. Also in the same year Nosotti used sternal cryopreserved allograft for reconstruction of chest wall after resection of chondrosarcoma of anterior thoracic cage [10]. In a report that was published by Dell'Amore et al., they described this method and their experiences in four patients that is the largest group of patients [8, 11, 12]. In Dell'Amore et al.'s series three of patients had malignant involvement of sternum and in one of them sternectomy was due to sternal infection after cardiac surgery [11]. Another successful experience in using sternal allograft was reported by Kaláb et al. in 2014 [13]. In all of these reports no complications that can be related to allograft have been reported. In our patient nearly one year after operation no complication has been observed.

Finally in this case we have reported two rare events occurring simultaneously. The first one is rare occurrence of aneurysmal bone cyst in flat bones especially sternum and the second is the use of a new and less described substitute after sternal resection. Ishinada et al. have described an aneurysmal bone cyst that was treated by total resection and reconstruction of defect by autogenous fibula [14]. Although some authors have described curettage of the tumor and filling the defect with bone graft or even radiotherapy as alternative modalities for treatment of aneurysmal bone cysts best treatment is complete surgical resection. Using sternal cryopreserved allograft for reconstruction of anterior chest wall after sternal resection is a simple and reproducible method. Although first published by Marulli et al. in 2010 [15], there are only limited number of patients that are treated by this technique and in all of them functional and cosmetic results, even in long term period, are satisfactory as in our patient. Also Marulli et al. in 2016 reported a case series that contained eighteen patients who underwent sternectomy and reconstruction by cryopreserved allograft. Although only in four patients total sternectomy was done, functional and cosmetic results were satisfactory [16]. More studies with larger number of patients and longer follow-up are needed to determine the long term result/outcome of this type of transplantation.
